# Reducing low birth weight: prioritizing action to address modifiable risk factors

**DOI:** 10.1093/pubmed/fdv212

**Published:** 2016-02-16

**Authors:** Christopher D Johnson, Siobhan Jones, Shantini Paranjothy

**Affiliations:** af1 Public Health Wales, Mold, Flintshire CH7 1PZ, UK; af2 Cardiff University School of Medicine, Heath Park, Cardiff CF14 4YS, UK

**Keywords:** low birth weight, modifiable risk factors, population attributable risk, pregnancy, tobacco

## Abstract

**Background:**

Low birth weight (LBW) affects 6.9% of all UK births and has remained largely unchanged for many years. The United Nations and the World Health Assembly have set targets to substantially reduce global incidence. Understanding the contribution of modifiable risk factors to the burden of LBW is required to ensure appropriate interventions are in place to achieve this reduction.

**Methods:**

Data from published studies on the risks from key modifiable factors were used alongside prevalence data from the Welsh population to calculate the population attributable risk for each factor individually and in combination.

**Results:**

Fourteen risk factors accounted for nearly half of LBW births, and 60% of those to younger mothers (<25 years). Tobacco smoke exposure was the largest contributor. We estimated that smoking in pregnancy was a factor in one in eight LBW births, increasing to one in five for women aged under 25.

**Conclusions:**

Risk factors are interrelated and inequitably distributed within the population. Exposure to one factor increases the likelihood of exposure to a constellation of factors further increasing risk. Action to address LBW must consider groups where the risk factors are most prevalent and address these risk factors together using multi-component interventions.

## Introduction

Approximately 53 000 UK live births (6.9%)^1^ were low birth weight (LBW) (born weighing <2500 g). Birth weight is inversely associated with infant mortality,^2^ and LBW is predictive of educational achievement,^3^ disability^2^ and diabetes, stroke and heart disease risk in adults.^2^ It is a key predictor of health inequalities and a key indicator of poverty. The United Nations General Assembly Special Session on Children targeted a reduction in LBW prevalence of one-third between 2000 and 2010,^4^ and the World Health Assembly endorsed targets to reduce LBW prevalence by 30% by 2025.^5^ LBW is considered a major factor in achieving Millennium Development Goal 4 on reducing infant mortality. However, LBW in the UK increased by 3% between 1980 and 2010.^1^

A number of risk factors are understood to cause LBW.^2^ Smoking in pregnancy is emphasized as a significant modifiable risk factor. The population attributable risk (PAR) from maternal smoking is estimated to be between 10 and 27%;^6^ however, this is directly related to maternal smoking prevalence and varies according to local prevalence.^6^ Inversely, 73–90% of LBW births are consequently attributable to other factors. It is therefore important to understand the impact of all modifiable factors.

Key risk factors from studies published up to December 2006, with outcomes of LBW, were examined in a 2008 report by the Institute of Health Economics (IHE).^2^ One hundred and thirty-one additional studies published between January 2007 and December 2013, estimating effect sizes for risk factors identified in the IHE report, were reported by Public Health Wales.^7^

This article uses these effect sizes alongside prevalence estimates to estimate the burden of LBW that is modifiable at the population level, and how this burden varies according to maternal age, using Wales as an example.

## Methods

A three-phase approach estimated the PAR for a range of modifiable risk factors.

### Data on effect sizes for risk factors

The articles included contained a relative risk (RR) or odds ratio (OR) of an LBW outcome associated with risk factors considered plausible and acceptably modifiable at a population level as a major finding of the study. Judgements on population modifiability were wider than individuals (e.g. young maternal age is modifiable by reducing teenage pregnancy) but acknowledged that some factors are not acceptably modifiable (e.g. discouraging older parenthood). Some factors had an unclear biological pathway and high risk of confounding with other known factors making causality implausible.

Effect size estimates were selected using the following hierarchy

- Pooled results from a number of studies in published meta-analyses.
- Larger or more recent studies with good evidence of control of confounders.
- Smaller, older or lower quality studies.

### Estimation of prevalence in Wales

Prevalence data were searched pragmatically with selected values based on a hierarchy of sources:

- Welsh Government or UK Government statistics specific to Wales
- UK Government Published Statistics specific to UK, England or Scotland
- English or Scottish Regional Statistics from Government Bodies
- Cohort or cross-sectional research studies based in the UK
- Combinations of the above

Preference was given to data on pregnant populations compared with the general population. Prevalence in under-25s often required application of age-specific rates or RRs to the proportion of babies born to mothers in specific age bands.

### Application of effect size and prevalence data to calculate attributable risks

The proportion of LBW births avoidable by removing exposure contributory factors was calculated for the general population and those aged under 25 years.

As LBW prevalence across the UK is <10%,^1^ we assumed the ‘rare disease assumption’ to hold and considered published ORs to be equivalent to RRs.

Population attributable fraction (PAF_e_) for individual risk factors was estimated from the relative risk (RR_e_) and the proportion of the population exposed (*P*_e_) (Equation 1).^8^

(1) $$\text{PA}{\text{F}}_{e}\;=\;\frac{P_{e}(R{\text{R}}_{e}\;-\;\text{1)}}{(P_{e}(R{\text{R}}_{e}\;-\;\text{1)) + 1}}\;$$

Not all risk factors identified could be studied in detail; therefore, we included only factors that had a PAF estimate >0.5%.

The combined effect of risk factors is not additive as overlap means the outcome can be attributed to more than one risk factor. The combined PAF was estimated as the product of one minus each individual PAF (Equation 2).^8^

(2) $$\text{combined }\;\text{PAF}=1-(\overset{n}{\underset{i=1}{\Pi }}(\text{1}\;-\;\text{PA}{\text{F}}_{e}))\;$$

PAFs are presented as PAR percentages, representing the proportion of LBW births preventable by removing the exposure.

### Selection of risk factors

Therefore, to be included as a factor needed to meet six criteria (Table 1).

- Being associated with LBW (<2500 g only) in a published study.
- Acceptably modifiable at a population level.
- Not currently screened for in pregnancy—Some infections are screened prenatally to reduce risk of adverse consequences in pregnancy.
- Plausible causal link to LBW.
- Equivalent prevalence data available—Some modifiable risk factors with important contributors had no comparable estimates of exposure prevalence.
- PAR estimates should be >0.5%.

**Table 1:** Risk factors included and excluded based on factors previously identified as having an association to low body weight^2,7^

*Risk factors*						
*Excluded*						*Included*
**No published RR or OR for LBW (<2500 g) outcome**	**Not considered acceptably modifiable at population level**	**Direct causality unlikely**	**No equivalent prevalence data**	**Screened in pregnancy**	**PAR <0.5% estimate**	**Hierarchy Group 1^a^** Smoking in pregnancy ETS exposure indoors Bacterial vaginosis Short inter-pregnancy interval Severe gum disease Chlamydia Cocaine Heroin/methadone Low BMI Anaemia Alcohol Hierarchy group 2^a^ Teenage pregnancy Cannabis intimate partner violence Hierarchy group 3^a^ None
Folic acid Vitamin D Urinary tract infections Air pollution	Long birth interval Previous history of LBW Maternal anatomical factors Infertility and IVF treatment Older maternal age Foetal factors	Minority race Unmarried parents Acculturation Biracial couples Unintended pregnancy	Adverse psychosocial factors Traffic density (proxy for air pollution) NOx CO SO_2_ Occupational factors	Syphilis HIV	Malaria Trichomoniasis Gonorrhoea Indoor air pollution	

^a^Risk estimate available from—Group 1: published meta-analyses; Group 2: Large or recent study with good evidence of control of confounders; Group 3: Small, older or lower quality studies.

## Results

### Risk factors and effect sizes

Seventy-three studies covering 14 risk factors including tobacco smoke, substance misuse, infections and nutritional factors met the inclusion criteria (Table 2).

**Table 2:** Prevalence and effect size estimates for modifiable risk factors for LBW ranked by their effect size

*Risk factor*	*Effect size*			*Prevalence*		
	*Risk range*	*Selected RR/OR*	*Source^a^*	*Population prevalence (%)*	*<25-year-old prevalence*	*Source*
Heroin/methadone	1.74–4.61	3.28	9	2	No data	10
Cocaine	2.15–4.42	2.85	11	1	No data	12
Smoking in pregnancy	1.43–2.00	1.9	13	16	28%	14
Severe gum disease	1.5–1.8	1.8	15	2^b^	Trace^b^	16
Cannabis	0.7–1.7	1.7	17	6.4	13.5%^b^	18
Low BMI	1.64–1.7	1.64	19	3	7%	20
Inter-pregnancy interval (1–5 m)	1.06–3.54	1.61	21	2	2.2%	22,23,24
Intimate partner violence	1.5–1.53	1.53	25	5	No data	26
Chlamydia	0.19–1.52	1.52	27	5	9%	28
Bacterial vaginosis	1.43–2.02	1.43	29	14.5	22.6%	30
Anaemia	1.29–1.94	1.29	31	24	30%	32
ETS exposure indoors	1.22–1.38	1.32	33	24	34%^b^	34
Teenage pregnancy	1.1–2.9	1.17	35	7	22.9%	24
Inter-pregnancy interval (6–11 m)	1.06–3.54	1.14	21	5.9	4.9%	22,23,24
Inter-pregnancy interval (12–18 m)	1.06–3.54	1.06	21	8.1	6.2%	22,23,24
Alcohol	0.64–2.67	1.06	36	39	30%	14

^a^A hierarchy of sources was used in effect size selection. Source listed is for selected study only.

^b^General population studies were used as a source of prevalence data for these risk factors as no suitable studies during pregnancy were available.

Active smoking^13,37–41^ and environmental tobacco smoke (ETS) exposure^33,38,42–44^ in pregnancy consistently increased the risk of LBW. Risks were higher for active smoking than for ETS exposure, which is consistent with strong evidence of a dose–response relationship for tobacco smoke and LBW.

Substance misuse including alcohol and illicit drugs increased the risk of LBW.^2^ Drinking alcohol increased risk of LBW compared with abstinance.^31,36^ Alcohol has a clear dose–response relationship with LBW^45^ with risk rapidly increasing with increased consumption, consequently drinker versus non-drinker assessments may be over-simplistic. Most women who drank throughout pregnancy reported consumption of <1 unit per week,^14^ which studies suggest has a minimal effect.^31^ Self-reported findings are likely to underestimate the true exposure, consequently, it is arguably best to base impact on a dichotomous drank/did not drink status. Cannabis use increased risk, although disagreement exists about its statistical significance.^17,46–49^ The estimate selected was from a large recent study which adequately controlled for alcohol, smoking and other illicit drugs and showed a significant effect.^17^ Use of heroin, methadone and cocaine showed the highest risk,^9,11,50^ suggesting those using illicit drugs in pregnancy or in harm reduction programmes are at especially increased risk.

Healthy weight and nutrition affect pregnancy outcomes. Substantial focus is placed on high body mass index (BMI), which was not linked to increased risk of LBW.^51,52^ However, low BMI is associated with increased risk of having a LBW baby.^19,51^ Anaemia is also a cause of LBW.^31,53,54^ A linear dose–response relationship between LBW risk and dietary iron exists with risk decreasing 3% for every 10 mg additional iron intake.^31^ Intervention trials show at 20% reduction in risk associated with supplementation,^55,31^ and a causal link is considered plausible.^8^

Infections including sexually transmitted infections, other non-STI genital infections and oral infections^2^ before or during pregnancy increase LBW risk. Individual studies examining Chlamydia infection were inconclusive.^56–59^ However, a meta-analysis reported a statistically significant increase in risk of LBW.^27^ Bacterial vaginosis, which is commonly experienced in pregnancy, and periodontal infections (gum disease and gingivitis) are also associated with increased risk of LBW.^15,29,60–62^

Maternal age has a U-shaped relationship with LBW displaying increased risk at both extremes and is an independent risk factor especially for the youngest mothers.^2^ A range of risks has been published especially for the youngest mothers;^35,43,63–68^ however, risk is increased for all teenagers. In Wales, only a small proportion of teenage mothers are under 16, consequently the most appropriate estimate was based on mothers aged <20.^35^ Inter-pregnancy interval also has a U-shaped relationship with lowest risk at an interval of 18–24 months.^21^ Intimate Partner or Domestic Violence is also a risk factor for LBW.^2,25,69^ Two systematic reviews and meta-analyses presented similar findings estimating a 50% increased risk for those exposed.^25,69^

### Estimation of prevalence in Wales

The prevalence of risk factors was estimated using the hierarchy outlined (Table 2). Prevalence for under-25s could not always be estimated or did not use using the highest ranking source. There were no published estimates of age-specific prevalence of cocaine and heroin use. Some evidence suggested that lifetime occurrence of intimate partner violence (IPV) is more prevalent in older age categories;^70,71^ however, measuring lifetime experience may skew results as older women have more years at risk or may be more confident to admit historical experience of domestic violence. The general population prevalence was therefore applied to under-25s for these factors.

ETS, periodontal infections and cannabis prevalence required age-specific rates which were available only from general population studies rather than from studies conducted in pregnancy to determine prevalence in under-25s group.

ETS exposure in pregnancy is measured for all pregnant women by the infant feeding survey (IFS).^14^ The Welsh Health Survey (WHS) publishes age-specific rates for exposure to tobacco smoke indoors,^34^ but these were not restricted to pregnancy. All persons aged 16–44^34^ were consistent with the reported prevalence in the IFS for all non-smoking mothers exposed to ETS,^14^ so WHS data were used for both age groups.

Periodontal infections in women were estimated by the 1999 Adult dental health survey (ADHS) showing a strong age gradient and low prevalence in women of child-bearing age (aged 24–44 = 2–3%, aged <24 = <1%).^16^ A pregnancy-specific cohort study estimated prevalence to be much higher (7.2%); however, the authors recognized that pregnancy hormones can lead to false positives.^72^ Consequently, the prevalence estimates for adult women aged under 44 from the ADHA were used.^16^

The National Crime Survey (NCS) showed that cannabis use was age dependent (6.4%—all adults versus 13.5% in 16–24 years old).^18^ Pregnancy-specific research studies produced varied estimates of cannabis use (5–15%),^12,18,73–75^ although participants did not always represent the whole population. The Avon Longitudinal Study of Parents and Children (ALSPC) used self-reported data from 12 000 participants to estimate cannabis use prevalence in pregnancy at 6%.^73^ This supports the NCS estimates suggesting little difference between Cannabis use in pregnancy and the general population. Consequently, the NCS was considered a good proxy for exposure in pregnancy in both age groups.^18^

Inter-pregnancy interval used a composite of sources to estimate prevalence. A large Scottish study recorded age-specific inter-pregnancy interval rates.^23^ Official statistics for 2013^22,24^ provided the proportion of all births with older siblings in each age group and age-specific rates for the intervals were then applied to these. Prevalence was lower in the under-25s on account of a lower proportion of births with a sibling; however, this masks that babies born to younger mothers who already have at least one child are far more likely to have very short birth intervals (Intervals <6 months—24% in under-20s versus <5% in 20–35 year olds).

Only alcohol use and periodontal infections showed a direct association with age where increasing age increased exposure,^14,16^ resulting in a lower prevalence in younger adults than for the population as a whole.

### Population attributable risks in Wales

For most risk factors, PAR was highest in younger mothers (age <25 years) (Table 3). Nearly half of all LBW births rising to nearly 60% to mothers aged under 25 could be prevented by removing exposure to these 14 risk factors.

**Table 3:** PARs for low birth weight from modifiable risk factors pregnant women of all ages and those under 25

*Risk factor*	*PAR*	
	*Population (%)*	*Under 25 years (%)*
Smoking in pregnancy	12.8	20.3
ETS exposure indoors	7.1	9.8
Anaemia	6.5	8
Bacterial vaginosis	5.9	8.9
Heroin/methadone	4.4	^a^
Cannabis	4.3	8.6
Inter-pregnancy interval	2.9	3.3
Chlamydia	2.5	4.6
Intimate partner violence	2.4	^a^
Alcohol	2.3	1.8
Cocaine	2	^a^
Low BMI	1.8	4.3
Severe gum disease	1.6	0
Teenage pregnancy	1.1	3.7
Totals	45.1	57.8

^a^Assumed to be identical to general population as no age-specific prevalence data available.

Maternal tobacco smoke exposure is the largest modifiable cause of LBW in Wales and the principal contributor to inequalities. One in eight LBW births are attributable to active smoking rising to one in five for mothers under age 25 years. For non-smokers, exposure to ETS is responsible for the highest proportion.

Poor nutrition, especially in younger mothers, also contributes substantially as evidenced by the contribution of anaemia and low BMI. Substance misuse is also a major contributor, and although prevalence of heroin and methadone use is low, the magnitude of the increased risk ensures these substances are among the largest contributors to the burden of LBW.

Sexual health also plays a key role. Bacterial vaginosis, Chlamydia and teenage pregnancy are responsible for substantial inequalities. One in 30 LBW births could be prevented by increasing birth interval, with slightly more preventable in younger age groups.

## Discussion

### Main findings of this study

The modifiable risk factors examined could be responsible for nearly half of all LBW births. The most striking finding is that most risk factors are more prevalent in younger mothers. Consequently, the proportion of preventable LBW births in this group is much higher. Limitations in the approach introduce uncertainty around the magnitude of the total burden attributable to modifiable risk factors. However, these factors contribute substantially and more so in younger mothers.

**Fig. 1:**
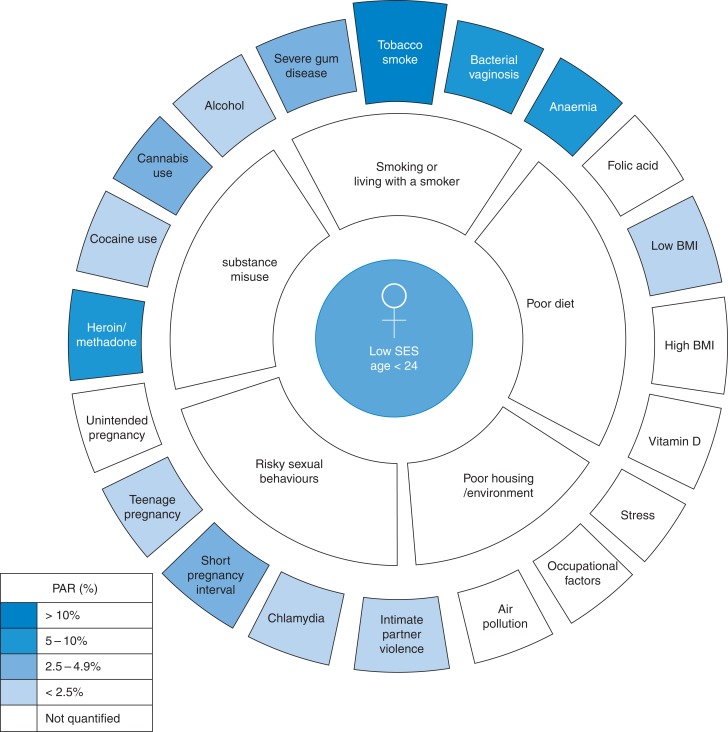
Grouping of individual risk factors around behaviours which place the largest burden of risk on younger mothers.

Individual risk factors can be grouped together and associated with key determinants that need to be addressed if substantial improvements are to be achieved (Fig. 1) and these cluster around the youngest mothers and the most deprived communities.

**Fig. 2:**
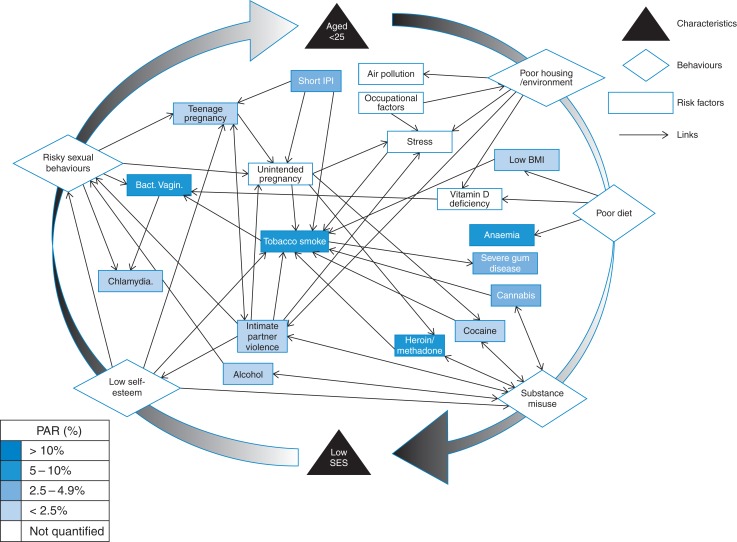
Relationship web between risk factors for LBW.

Moreover, the risk factors cannot be addressed in isolation. Many of these risk factors, although independently associated with LBW are also strongly linked to each other. Bacterial vaginosis is more common in smokers, those with vitamin D deficiency and those with multiple sexual partners.^30,76,77^ Infection with BV increases susceptibility to developing Chlamydia.^78^ Substance misuse and tobacco use are strongly associated.^72^ Additionally IPV, unintended pregnancy and substance misuse are associated with LBW and each other.^79^ The overlapping links identified existing between risk factors and behaviour groups (Fig. 2 and Supplementary data, Table S1) demonstrate that women, and most likely those under 25, are affected by constellations of risk factors and that addressing them individually will not achieve the desired effect.

The most influential independent factor and the one most linked to other risks is tobacco smoke exposure. Even if maternal smoking was reduced to zero in pregnancy, the exposure to partners and family members smoking ensures tobacco smoke remains the largest independent modifiable cause of LBW. Consequently, risk factors must not be addressed solely at an individual level, but at a household, family and community level. Reducing both smoking and environmental tobacco exposure in pregnancy and to children in early years must be the main public health priority if this issue is to be addressed.

It has been estimated that the cost to the NHS of delivering LBW babies is on average £1993 more expensive than non-LBW births^80^ when converted to 2013 values.^81^ Two thousand four hundred LBW births occur in Wales.^82^ Removing exposure to modifiable risk factors and concomitant reduction in LBW could save NHS maternity services an estimated to be £2.15million on the additional cost of LBW births alone based on PARs in this study. Expanded across the UK, this means over 24 000 preventable LBWs costing the NHS £49 million.

### What is already known?

Existing literature identified a wide range of risk factors for LBW and produced wide ranging effect size estimates. The quality of these studies and the outcomes they report are varied. However, for many factors it has allowed good estimation of the magnitude of the risk. The prevalence of some risk factors in the UK is also known either from cohort studies or from official statistics, although not in every case. Despite this data being available, the overall contribution of the risk factors to LBW in Wales or the UK has not been previously explored.

### What this study adds?

This study combined evidence on modifiable risk factors and their prevalence in Wales to quantify the impact on LBW. It has shown how these risk factors cluster and form constellations around individuals. Young mothers in deprived communities are more likely to be exposed to most of the other modifiable risk factors as well. The magnitude of the health inequalities experienced by younger mothers and their babies is shown with LBW much more likely. It must be remembered that those under 24 are nearly 25% of the general population, and therefore, the gap between those most at risk (generally <24 in lower SES categories) and those least at risk (generally >30 professional women) will be even greater.

### Limitations of the study

The data used to estimate prevalence is based on a cornucopia of disparate sources examining populations with varied location and age, adding uncertainty to the estimates and to whether they are representative of Wales or any population. This is likely to be a non-systematic error that will lead to random under or over estimation of prevalence for each risk factor.

Additionally, the study is unable to examine the links between the risk factors to the extent desired. Even when grouped, the PAR estimates still consider the risk factors independently. The true increased risk for an individual exposed to numerous risk factors is unclear. The underlying assumption is that influence is additive, each additional factor contributes the same additional risk it would independently. The model assumes factors overlap but do not influence. We know these influences exist, e.g. a cannabis user is more likely to smoke, and consequently is more likely to develop BV, etc., through the chain. The effect is a systematic bias, but it is difficult to determine whether risk is systematically under- or over-estimated.

We acknowledge that this methodology resulted in the exclusion of some risk factors with good evidence of an association with LBW due to insufficient evidence quantifying this effect, but their contribution should not be ignored.^7^ Robust objective estimates exist for a limited number of factors and important risk factors (e.g. Vitamin D and air pollution) were unquantifiable, because effect size or prevalence data in the correct format were not available. For birth interval, no detailed official statistics are published, only medium interval, and so estimates have to be extrapolated from several sources. The cornucopia of different outcomes used, e.g. LBW, small of gestational age, mean birth weight reduction, etc., limit data availability and make a judgement on the effect size challenging as terminology is not comparable. It becomes difficult to prove what the weight of evidence suggests and contributions from risk factors (e.g. vitamin D deficiency) cannot be demonstrated. Being unable to quantify factors that make substantial contributions to LBW, and are not independent of those quantified, leads to systematic under-estimation of impact.

To address these limitations, more research into the prevalence of the main risk factors in Wales and the extent to which factors influence each other is needed. A better understanding of how factors combine to increase risk is required. The calculations are underpinned by assumptions of independence and causality. Consequently, risks are assumed to be additive, and factors combine randomly. We know this is not the case. The risk factors are identified from observational studies and may not be causal or may share causal pathways. To fully understand the extent of the burden on healthcare and health inequalities these modifiable risk factors cause through LBW, we need to fully understand the interactions.

## Conclusion

The majority of LBW is avoidable by addressing the modifiable risk factors. Parts of our communities are hit hardest by this burden, and it is possible to see that young women are caught within constellations of factors. A change of approach is vital, moving from addressing individual risk factors with individuals in isolation to addressing co-occurring groups of factors with the whole family, household and community around the women most at risk.

## Supplementary data

Supplementary data are available at the *Journal of Public Health* online.

## Supplementary Material

Supplementary Data
