# Supplementary material for: Reducing low birth weight: prioritizing action to address modifiable risk factors
Source: J Public Health (Oxf). 2016 Feb 16;39(1):122–31. doi: 10.1093/pubmed/fdv212 (PMC5409066; doi:10.1093/pubmed/fdv212)
Supplement: Supplementary Data [file supplementary_table_1.docx]

Supplementary table 1: Evidence for links in the relationship web between risk factors for low birth weight

| **Relationship** | | **Evidence** | |
| --- | --- | --- | --- |
| **Behaviour / Risk Factor** | **Linked Risk Factor** | **Description of link** | **Citation** |
| Risky Sexual Behaviour | Teenage Pregnancy | UK NATSAL-3 study carried out between 2010-2012 found that pregnancies in women aged 16-19 were the most commonly unplanned (45%) or ambivalent (43%) and least often planned (12%). | Wellings K. *et al*. The prevalence of unplanned pregnancy and associated factors in Britain: findings from the third National Survey of Sexual Attitudes and Lifestyles (Natsal-3). The Lancet. 2013. 382(9907) 1807:1816 |
|  | Unintended Pregnancy | UK NATSAL Study carried out in 2010-11 found that odds of having an unplanned pregnancy increased in those with higher frequency of sex (>4 times in previous week) and more than one sexual partner in the last year. | Wellings K. *et al*. The prevalence of unplanned pregnancy and associated factors in Britain: findings from the third National Survey of Sexual Attitudes and Lifestyles (Natsal-3). The Lancet. 2013. 382(9907) 1807:1816 |
|  | Bacterial Vaginosis | Bacterial Vaginosis has been shown to be more prevalent in women who have a history of multiple sexual partners or previous STI infection. | Morris M, Nicoll A, Simms I, Wilson J, Catchpole M. Bacterial Vaginosis: a public health review. Br J Obstet Gynaecol. 2001; 108: 439-450.  Oakeshott P et al.. Association between bacterial vaginosis or chlamydial infection and miscarriage before 16 weeks' gestation: prospective community based cohort study. Br Med J. 2002; 325(1344). |
|  | Chlamydia | UK NATSAL study carried out in 2010-11 identified higher numbers of partners especially without condom use as risk factors for Chlamydia infection. | Sonnenberg, Pam et al. Prevalence, risk factors, and uptake of interventions for sexually transmitted infections in Britain: findings from the National Surveys of Sexual Attitudes and Lifestyles (Natsal) The Lancet , 382(9907) , 1795 - 1806 |
| Low Self Esteem | Risky Sexual Behaviours | There is evidence that high self-esteem can have an reduce risk risky sexual behaviours in teenagers. Several studies have indicated that high self esteem is linked to higher contraception use. | Emler, N. Self-esteem: The costs and causes of low self-worth. Joseph Rowntree Foundation. York, 2001 |
|  | Teenage Pregnancy | There is evidence that the risk of pregnancy in teenage girls with low self esteem is as much as 50% higher than in those with higher self esteem. | Emler, N. Self-esteem: The costs and causes of low self-worth. Joseph Rowntree Foundation. York, 2001 |
|  | Tobacco Smoke Exposure | The millennium cohort study of over 18000 women in the UK showed that low self-esteem was more prevalent in heavy smokers than those that quit or never smoked. | Pickett, K E, Wilkinson, R G and Wakschlag, L S, The psychosocial context of pregnancy smoking and  quitting in the Millennium Cohort Study. J Epidemiol Community Health 2009;63:474–480 |
|  | Substance Misuse | There have been a large number of studies linking low-self esteem to increased risks of substance misuse, using both alcohol and illicit drugs. Although it is difficult to determine the direction of the link with both low self esteem increasing risk of substance misuse, and substance misuse lowering self esteem being equally plausible. | Emler, N. Self-esteem: The costs and causes of low self-worth. Joseph Rowntree Foundation. York, 2001 |
| Substance Misuse | Alcohol Use | Alcohol, heroin, methadone, cocaine and cannabis are all considered substances which full under the definition of substance misuse. | |
|  | Heroin Methadone Use |  |  |
|  | Cocaine Use |  |  |
|  | Cannabis Use |  |  |
| Poor diet | Anaemia | A number of dietary factors including lack of iron, folic acid and vitamin B12 are all associated with anaemia in pregnant and non-pregnant women. | Rasmussen K. Is there a causal relationship between iron deficincy or iron-deficiency anaemia and weight at birth, length of gestation and perinatal mortality. J Nutr. 2001; 131(2S-2): 590S-601S. |
|  | Vitamin D deficiency | Vitamin D is sourced through access to sunlight and to oily fish in the diet. Increased oily fish consumption decreases the risk of vitamin D deficiency. | Holick, MF. Vitamin D deficiency. N Engl J Med 2007; 357:266-281 |
|  | Low BMI | The link between poor quality or insufficient dietary intake of calories and other nutrients and subsequent body weight is self evident. | |
| Poor housing / Environment | Vitamin D deficiency | A study in Scotland correlated time spent on outdoor activities with the likelihood of vitamin D deficiency. Outdoor activities included gardening, walking and cycling.  Poor quality urban environments which are not conducive to spending time outdoors will therefore increase risk. | Zgaga, L *et al. Diet,* Environmental Factors, and Lifestyle  Underlie the High Prevalence of Vitamin D  Deficiency in Healthy Adults in Scotland, and  Supplementation Reduces the Proportion That  Are Severely Deficient. Journal of Nutrition, 2011, 141(8), 1535-42 |
|  | Stress | Poor or overcrowded housing is a known environmental chronic stressor.  Noise and run down built environments (e.g. graffiti) can also contribute to stress. | Kramer, MS *et al.* Socio-economic disparities in pregnancy outcome. Why do the poor fare so poorly? *Paediatric and perinatal epidemiology*. 2000, 14, 194-210  Glaster, G. The Mechanism(s) of Neighbourhood Effects Theory, Evidence, and Policy Implications. *Neighbourhood effects research: New perspectives.* 2012. Springer, London |
|  | Air Pollution | There is a large body of evidence linking low-income housing to increased air pollution. | Glaster, G. The Mechanism(s) of Neighbourhood Effects Theory, Evidence, and Policy Implications. *Neighbourhood effects research: New perspectives.* 2012. Springer, London |
|  | Intimate Partner Violence | The Avon longitudinal survey found that both housing defects and inadequate housing were both statistically significantly associated with a intimate partner violence in pregnancy. | Bowen, E *et al* Domestic violence risk during and after pregnancy: findings from a British longitudinal study. BJOG, 2005; 112, 1083–1089, |
| Short Interpregnancy Interval | Tobacco Smoke Exposure | A study of 69,000 women in Scotland found that women with short interpregnancy intervals were more likely to smoke than those with longer intervals. | Smith GCS, Pell JP, Dobbie R. Interpregnancy interval and risk of preterm birth and neonatal death: retrospective cohort study. Br Med J. 2003; 327. |
|  | Teenage Pregnancy | A study of 69,000 women in Scotland found that women with short interpregnancy intervals were more likely to be aged less than 20 than those with longer intervals. | Smith GCS, Pell JP, Dobbie R. Interpregnancy interval and risk of preterm birth and neonatal death: retrospective cohort study. Br Med J. 2003; 327. |
|  | Unintended Pregnancy | A study of 11,000 pregnancies in Denmark showed a significantly higher odds of a short interval if the pregnancy was unplanned (OR=2.9, 95% CI: 2.2–3.9) | Kaharuza, F. M., Sabroe, S. and Basso, O. (2001), Choice and chance: Determinants of short interpregnancy intervals in Denmark. Acta Obstetricia et Gynecologica Scandinavica, 80: 532–538. |
| Teenage Pregnancy | Unintended Pregnancy | A study of over 5000 pregnant women in booking for abortion or prenatal care in Edinburgh found that unintended pregnancy whether continued or aborted was significantly associated with age, younger women being more likely to have not intended to become pregnant. | Lakha, F and Glasier A. Unintended pregnancy and use of emergency contraception among a large cohort of women attending for antenatal care or abortion in Scotland. The Lancet. 2006. 368(9549). 1782:1787 |
|  | Intimate Partner Violence | A study of over 12,000 women in the USA found higher levels of intimate partner violence in teenagers. | Gazmararian JA, Adam M, Saltzman LE et al. The relationship between pregnancy intendedness and physical violence in mothers of newborns. Obstet Gynaecol. 1995; 85: 1031-1038. |
| Bacterial Vaginosis | Chlamydia | Subjects with Bacterial Vaginosis were 3 time more likely to also test positive for Chlamydia infection. Presence of hydrogen peroxide producing lactobacilli were believed to be protective against STI infection. | Wiesenfeld HC *et al*. Bacterial Vaginosis is a strong predictor of Neisseria gonorrhoeae and Chlamydia trachomatis infection. HIV/AIDS. 2003; 36: 663-668. |
| Unintended Pregnancy | Stress | A study of 2500 women in the USA found statistically increased risk of reporting stress in women whose pregnancy was unintended. | Messer LC *et al.* Pregnancy Intendedness, Maternal Psychosocial Factors and Preterm Birth. Maternal and Child Health Journal. 2005, 9(4), 403-412 |
|  | Tobacco Smoke Exposure | A study of 9000 women showed those with unwanted or mistimed pregnancies were significantly more likely to have unhealthy behaviours including smoking prenatally.  UK NATSAL Study carried out in 2010-11 found that odds of having an unplanned pregnancy were double in current smokers. | Cheng, D *et al* Unintended pregnancy and associated maternal preconception, prenatal and postpartum behaviours. Contraception, 2009, 79(3), 194-198  Wellings K. *et al*. The prevalence of unplanned pregnancy and associated factors in Britain: findings from the third National Survey of Sexual Attitudes and Lifestyles (Natsal-3). The Lancet. 2013. 382(9907) 1807:1816 |
|  | Heroin / Methadone.  Cocaine | UK NATSAL Study carried out in 2010-11 found that odds of having an unplanned pregnancy if the women used drugs other than cannabis were 3 times greater than those that did not.. | Wellings K. *et al*. The prevalence of unplanned pregnancy and associated factors in Britain: findings from the third National Survey of Sexual Attitudes and Lifestyles (Natsal-3). The Lancet. 2013. 382(9907) 1807:1816 |
| Intimate Partner Violence | Unintended Pregnancy | A review into the impact of IPV on pregnancy found a number of studies that link IPV to the likelihood of an unintended pregnancy due to a reduction in fertility control, even after control for socioeconomic factors.  Furthermore, women with mistimed or unwanted pregnancies have been shown to be at greater risk of violence than those whose pregnancy is intended. | Sarkar, N. N. The impact of intimate partner violence on  women's reproductive health and pregnancy  outcome. Journal of Obstetrics and Gynaecology, 2008. 28:3,  266-271  Gazmararian JA, Adam M, Saltzman LE et al. The relationship between pregnancy intendedness and physical violence in mothers of newborns. Obstet Gynaecol. 1995; 85: 1031-1038. |
|  | Low Self Esteem | Studies have shown that women who experience IPV during pregnancy are more likely to score lower on measures of self-esteem and social support. | Curry, MA *et al* , Stress related to domestic violence during pregnancy and infant birth weight.  1998, Empowering survivors of abuse: Health care for battered women and their children. Sage series on violence against women., (pp. 98-108). Thousand Oaks, CA, US: Sage Publications, Inc, xii, 332 pp. |
|  | Risky Sexual Behaviour | Silverman et al found Adolescent girls who reported abuse from dating partners were found to be at significantly elevated risk of engaging in sexual health risk behavior, including first intercourse before the age of 15 years and multiple partnering | Silverman J.G. *et al* Dating Violence Against Adolescent Girls and Associated Substance Use, Unhealthy Weight Control, Sexual Risk Behavior, Pregnancy, and Suicidality. JAMA. 2001;286(5):572-579 |
|  | Tobacco Smoke Exposure | A significant increased risk of current cigarette use was noted among pregnant women who were absused in a Canadian study of 4750 women. | Janssen P A *et al*, Intimate partner violence and adverse pregnancy outcomes: A population-based study. Am J Obstet Gynecol 2003;188:1341-7.) |
|  | Stress | Stress has been linked to IPV, and increased stress during pregnancy has can increase the risk of violence.  In a Canadian study of over 700 women found that stress and negative life events prior to pregnancy were both significantly associated with an increased risk of IPV. | Jasinski, JL: Pregnancy and domestic violence: a review of the literature. Trauma, Violence and Abuse, 20014, 5(1), , 47-64  Muhajarine, N and D’Arcy C. Physical abuse during pregnancy: prevalence and risk factors. CMAJ, 1999; 160 (7), 1007-1011 |
|  | Substance Misuse | Silverman et al found Adolescent girls who reported abuse from dating partners were found to be at significantly elevated risk of misuse of use alcohol, tobacco, and cocaine after controlling for the effects of confounding risk behaviours and demographics.  Janssen et al also noted that there was a significant risk of alchol and illict drug use among pregnant women who were absused. | Silverman J.G. *et al* Dating Violence Against Adolescent Girls and Associated Substance Use, Unhealthy Weight Control, Sexual Risk Behavior, Pregnancy, and Suicidality. JAMA. 2001;286(5):572-579  Janssen P A *et al*, Intimate partner violence and adverse pregnancy outcomes: A population-based study. Am J Obstet Gynecol 2003;188:1341-7.) |
|  | Teenage pregnancy | Silverman et al found Adolescent girls who reported abuse from dating partners were found to be at significantly elevated risk of having been pregnant | Silverman J.G. *et al* Dating Violence Against Adolescent Girls and Associated Substance Use, Unhealthy Weight Control, Sexual Risk Behavior, Pregnancy, and Suicidality. JAMA. 2001;286(5):572-579 |
| Heroin /Methadone Use | Tobacco Smoke Exposure | In an analytical study of 800 urine samples taken in early pregnancy screened for traces of illicit drugs and cotinine as an indicator of smoking status it was found that significantly higher proportion of illict drug users also had cotinine levels indicative of smoking than the non-drug group. | Sherwood R, et al Substance misuse in early pregnancy and relationship to fetal outcome. Eur J Paeds. 1999; 158: 488-492. |
| Cocaine Use |  |  |  |
| Cannabis Use |  |  |  |
| Tobacco Smoke Exposure | Severe Gum Disease | This study looked at dental examination of 2000 pregnant women presenting to a London hospital around 12 weeks duration for routine ultrasound scans. Loss of attachment and bleeding score showed a relationship with smoking status  during pregnancy. The relationship of  probing depth to smoking status approached significance (p=0.057). | Moore, S. *Et* al. Periodontal health of London women  during early pregnancy. British Dental Journal, 191, 570 - 573 (2001) |
|  | Bacterial Vaginosis | Subjects who smoked were 60% more likely to exhibit Bacterial Vaginosis than those that did not. Prevalence of bacterial Vaginosis amongst smokers was 17% compared to 11% in non-smokers. | Oakeshott P et al.. Association between bacterial vaginosis or chlamydial infection and miscarriage before 16 weeks' gestation: prospective community based cohort study. Br Med J. 2002; 325(1344).  Morris M, Nicoll A, Simms I, Wilson J, Catchpole M. Bacterial Vaginosis: a public health review. Br J Obstet Gynaecol. 2001; 108: 439-450. |
| Vitamin D Deficiency | Bacterial Vaginosis | Women with Vitamin D deficiency in pregnancy are nearly 3 times more likely to develop bacterial Vaginosis compared to those who are not deficient. | Hensel et al, Pregnancy-specific association of vitamin D deficiency and bacterial Vaginosis, Am J Obstet Gynecol 2011;204:41.e1-9 |
| Low BMI | Tobacco Smoke Exposure | Prevalence of smoking in underweight pregnant women (35.1%) was significantly higher than that observed in normal weight pregnant women (21.5%) in a sample of over 95,000 women from the UK. | Denison FC, *et al.* Association between maternal body mass index during pregnancy, short term morbidity, and increased health service costs: a population based study. BJOG. 2014; 121: 72-82. |
| Stress | Tobacco Smoke Exposure | The Millennium cohort study of over 18,000 women in the UK showed that women were much more likely to be heavy smokers if the displayed one or more problems in the psychosocial characteristics. | Pickett, K E, Wilkinson, R G and Wakschlag, L S, The psychosocial context of pregnancy smoking and  quitting in the Millennium Cohort Study. J Epidemiol Community Health 2009;63:474–480 |
| Occupational Factors | Poor housing / Environment | Links between occupation, housing, neighbourhood, poverty and mental and physical health inequalities has been well established. | |
|  | Stress |  |  |
| Alcohol Use | Risky Sexual Behaviour | Alcohol has been shown to reduce contraception use by sexually active teenagers. | Grossman, M and Markowitz S. I Did What Last Night?!!! Adolescent Risky Sexual Behaviors and Substance Use Eastern Economic Journal, 2005, 31(3), 383-405 |
